# Supplementary material for: Clinical Association between Gout and Parkinson’s Disease: A Nationwide Population-Based Cohort Study in Korea
Source: Medicina (Kaunas). 2021 Nov 24;57(12):1292. doi: 10.3390/medicina57121292 (PMC8704991; doi:10.3390/medicina57121292)
Supplement: Supplementary file 1 [file medicina-57-01292-s001.zip › medicina-1441654-supplementary.pdf]

Table S1. Cox proportional hazard model of risk factors for Parkinson's disease by sex.

	Male group Adjusted HR (95% CI)	P-value	Female group, Adjusted HR (95% CI)	P-value
Gout	0.96 (0.86-1.07)	0.4485	1.19 (0.96-1.47)	0.1176
Hypertension	1.12 (0.96-1.3)	0.1432	1.46 (0.96-2.22)	0.0793
Diabetes	0.95 (0.84-1.08)	0.4145	0.84 (0.65-1.1)	0.2036
Dyslipidemia	0.6 (0.52-0.69)	<.0001	0.59 (0.42- 0.82)	0.0017
Ischemic heart disease	0.98 (0.87-1.09)	0.6557	1.07 (0.85- 1.34)	0.5597
Stroke	2.15 (1.91 -2.41)	<.0001	1.39 (1.12-1.73)	0.0025

HR: hazard ratio (using the Cox proportional hazard model), CI: confidence interval
